# Improvement in the Anti-Tumor Efficacy of Doxorubicin Nanosponges in In Vitro and in Mice Bearing Breast Tumor Models

**DOI:** 10.3390/cancers12010162

**Published:** 2020-01-09

**Authors:** Monica Argenziano, Casimiro Luca Gigliotti, Nausicaa Clemente, Elena Boggio, Benedetta Ferrara, Francesco Trotta, Stefania Pizzimenti, Giuseppina Barrera, Renzo Boldorini, Federica Bessone, Umberto Dianzani, Roberta Cavalli, Chiara Dianzani

**Affiliations:** 1 Dipartimento di Scienza e Tecnologia del Farmaco, University of Torino, 10125 Torino, Italy; monica.argenziano@unito.it (M.A.); benedetta.ferrara@unito.it (B.F.); f.bessone@unito.it (F.B.); chiara.dianzani@unito.it (C.D.); 2 Department of Health Sciences and Interdisciplinary Research Center of Autoimmune Diseases (IRCAD), University of Eastern Piedmont (UPO), 28100 Novara, Italy; luca.gigliotti@med.uniupo.it (C.L.G.); nausicaa.clemente@med.uniupo.it (N.C.); elena.boggio@med.uniupo.it (E.B.); renzo.boldorini@med.uniupo.it (R.B.); umberto.dianzani@med.uniupo.it (U.D.); 3 Department of Chemistry, University of Torino, 10125 Torino, Italy; francesco.trotta@unito.it; 4 Department of Clinical and Biological Sciences, University of Turin, 10125 Torino, Italy; stefania.pizzimenti@unito.it (S.P.); giuseppina.barrera@unito.it (G.B.)

**Keywords:** β-cyclodextrin nanosponges, doxorubicin, breast cancer, BALB-neuT mice, EPR effect

## Abstract

Doxorubicin (DOX) is an anthracycline widely used in cancer therapy and in particular in breast cancer treatment. The treatment with DOX appears successful, but it is limited by a severe cardiotoxicity. This work evaluated the in vitro and in vivo anticancer effect of a new formulation of β-cyclodextrin nanosponges containing DOX (BNS-DOX). The BNS-DOX effectiveness was evaluated in human and mouse breast cancer cell lines in vitro in terms of effect on cell growth, cell cycle distribution, and apoptosis induction; and in vivo in BALB-neuT mice developing spontaneous breast cancer in terms of biodistribution, cancer growth inhibition, and heart toxicity. BNS-DOX significantly inhibited cancer cell proliferation, through the induction of apoptosis, with higher efficiency than free DOX. The breast cancer growth in BALB-neuT mice was inhibited by 60% by a BNS-DOX dose five times lower than the DOX therapeutic dose, with substantial reduction of tumor neoangiogenesis and lymphangiogenesis. Biodistribution after BNS-DOX treatment revealed a high accumulation of DOX in the tumor site and a low accumulation in the hearts of mice. Results indicated that use of BNS may be an efficient strategy to deliver DOX in the treatment of breast cancer, since it improves the anti-cancer effectiveness and reduces cardiotoxicity.

## 1. Introduction

Doxorubicin (DOX) is one of the most effective anticancer drugs used against solid tumors of diverse origins and in particular against breast cancer. However, its use is associated with severe adverse effects including cardiotoxicity, myelosuppression and palmar plantar erythrodysenthesia, which impose a narrow therapeutic dose limiting DOX effectiveness [1]. Moreover, cancer cells frequently become resistant to DOX treatment and some conditions of the tumor microenvironment-such as hypoxia, acidity, defective vasculature, and lymphatic vessels-limit the DOX anticancer effectiveness [2].

Hence, there is necessity for an effective approach that limits DOX side effects by reducing DOX doses and modifying its bio-distribution to favor its concentration into the tumor site. To this aim, we evaluated the possibility of incorporating DOX into nanoparticles capable of favoring accumulation of the drug into the tumor site. In recent years, the development of nano-delivery systems for anticancer agents, with dimensions of less than 1000 nm have been found to be acceptable for intravenous administration, and have improved the therapeutic effectiveness of several compounds. Compared to conventional carriers, they report several advantages, such as capacity to solubilize hydrophobic drugs, reduction of total drug dose, and prevention of side effects [3]. When infused in the bloodstream, nanoparticles can accumulate in tumors owing to the enhanced permeability and retention (EPR) effect since the immature vasculature of tumors display fenestrations smaller than 200 nm, allowing for extravasation and entrapment of nanoparticles from the blood into the tumor tissue [4]. Recent studies showed that DOX and mitomycin C, co-loaded in polymer–lipid hybrid nanoparticles, inhibited the growth of sensitive and multidrug resistant human mammary tumor xenografts [2].

Many cyclodextrin (CD) derivatives have been proposed for drug delivery, and they have been shown to improve bioavailability, aqueous solubility, and stability of hosted drugs [5,6,7,8]. β-cyclodextrin nanosponges (BNS) are hyper-cross-linked polymers with CD units as the building blocks of the system and are a biocompatible and suitable nanosystem to carry drugs [9,10,11]. Different anticancer drugs-such as paclitaxel, camptothecin, and tamoxifen-have been efficiently incorporated in nanosponges, showing an improved antitumor effect [12]. In this study, DOX was loaded in β-cyclodextrin based nanosponges (BNS-DOX), and the effect of DOX and BNS-DOX was compared in vitro in human and mouse breast cancer cell lines, either sensitive or resistant to DOX, and in vivo in the growth of spontaneous mammary cancer in BALB-neuT mice, which mimics some of the most critical features of the human disease and represents a real model of human epidermal growth factor receptor 2 (HER2) positive breast cancer [13]. Results indicated that use of BNS may be an efficient strategy to deliver DOX in the treatment of breast cancer, since it improves the anti-cancer efficacy and reduces cardiotoxicity.

## 2. Results

The in vitro and in vivo results are listed below. Table 1 reports the physico-chemical characteristics of blank BNS and BNS loaded with DOX. BNS nanoformulations with an average diameter of about 300 nm and negative surface charge were obtained. Both the BNS nanoformulations displayed similar sizes. Doxorubicin was incorporated in the nanosponge nanostructure in a good extent. Indeed, the loading capacity and encapsulation efficiency of BNS-DOX were 16.4% and 98.5%, respectively. Notably, the drug loading did not affect the physico-chemical parameters of BNS and the physical stability of the nanosuspensions. The physical stability of BNS-DOX stored at 4 °C was confirmed up to 1 month.

**Figure 1:**
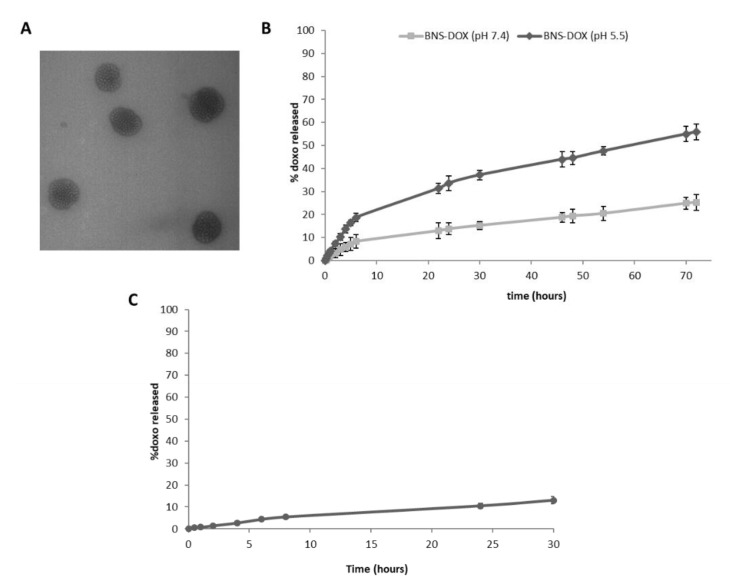
Characterization of β-cyclodextrin nanosponges containing doxorubicin (BNS-DOX). (**A**) Transmission electron microscopy image of BNS-DOX (scale bar 150 nm). (**B**) In vitro release kinetics of DOX from BNS-DOX at the two different pH levels (5.5 and 7.4). (**C**) In vitro release kinetics of DOX from BNS-DOX in rat plasma at 37 °C.

The spherical morphology of BNS-DOX and their nanometric sizes were shown by Transmission electron microscopy (TEM) analysis (Figure 1A).

DOX was released from BNS-DOX with a prolonged release kinetics, without initial burst effect (Figure 1B). This in vitro release profile confirmed the DOX incorporation in BNS polymeric matrix. The percentage of DOX released was higher at acidic pH values. Indeed, it increased about 2.5 fold at pH 5.5 compared to that at pH 7.4 (Figure 1B). The slow and prolonged in vitro release profile of DOX from BNS-DOX was observed also in plasma and it was comparable to that of the one obtained in phosphate buffered saline (PBS) at pH 7.4 (Figure 1C). After 24 h, the percentage of DOX released from the BNS was 10.5% and 13% in plasma and in buffer (pH 7.4), respectively.

No changes in the BNS physico-chemical parameters and no aggregation phenomena were observed after incubation in plasma at 37 °C for 24 h, confirming the BNS-DOX stability.

In order to determine the BNS-DOX effect in tumor cell growth, (3-(4,5-Dimethylthiazol-2-yl)-2,5diphenyltetrazolium bromide (MTT) analysis was performed in breast tumor cells lines, which were human MDA-MB231 and MCF-7 cells (both DOX-sensitive), and mouse 4T1 (DOX-sensitive) and EMT6/AR10r (DOX-resistant) cells. MDA-MB231, 4T1, and EMT6/AR10r were chosen as models of triple negative tumors (TNBC)-i.e., negative for estrogen receptor (ER), progesterone receptor (PR), and HER2-while MCF-7 was chosen as a model of ER/PR positive tumors.

**Figure 2:**
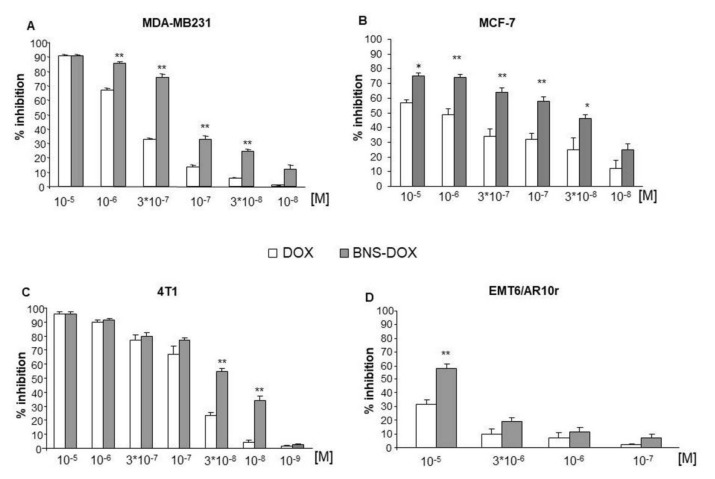
Percentage of cell survival following DOX and BNS-DOX treatment. (**A**,**B**) MDA-MB231 and MCF-7 human breast cancer cell lines and (**C**,**D**) 4T1 and EMR6/AR10r mouse breast cancer cell lines were treated with increasing concentrations of the drugs for 72 h. Results are expressed as % of viability inhibition of control shown as mean ± SEM (*n* = 5). Eight replicate wells were used to determine each data point, and five different experiments were performed. * *p* < 0.05, ** *p* < 0.01, significantly different from the same concentration of DOX.

To assess the effect on cell viability, cells were cultured in the presence and absence of titrated concentrations (10^−5^–10^−9^ M) of DOX or BNS-DOX and the MTT assay was performed after 72 h. Results showed that BNS-DOX inhibited cell viability in a concentration-dependent manner in all cell lines with higher efficacy than DOX (Figure 2).

In particular, EMT6/AR10r cells were quite resistant to both drug formulations and only the highest dose of BNS-DOX (10^−5^ M) substantially inhibited the cell growth. The empty BNS did not show any level of toxicity, even at high concentrations. Table 2 reports the half maximal inhibitory concentration (IC_50_) obtained from these experiments and shows that BNS-DOX displays lower IC_50_ than DOX in all cell lines.

Results show that the inhibitory effect was exerted by BNS-DOX at a concentration always lower (3–10 times lower) than free DOX. Since a main limitation of DOX is its cardiotoxicity, we performed the same experiments on rat cardiomyocyte H9c2 cells, but results showed that BNS-DOX and DOX did not display a substantial difference in inhibiting cell viability of these cells.

**Figure 3:**
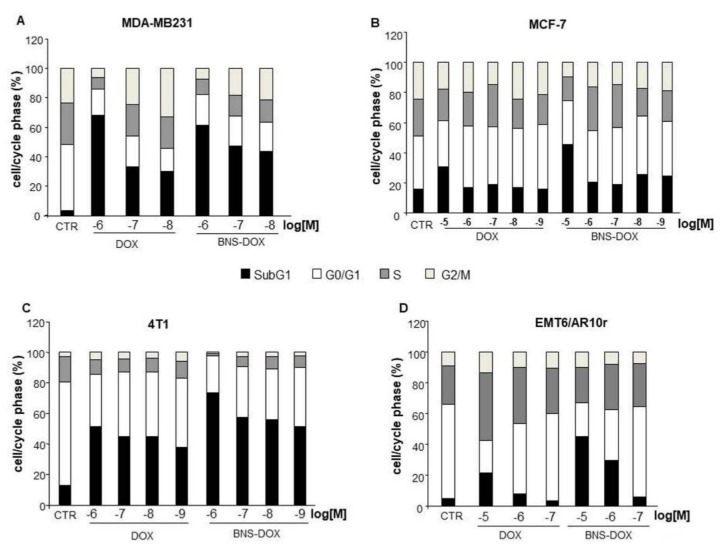
Effects of DOX and BNS-DOX treatment on cell cycle. (**A**,**B**) MDA-MB231 and MCF-7 human breast cancer cell lines and (**C**,**D**) 4T1 and EMR6/AR10r mouse breast cancer cell lines (1.5 × 10^5^) were treated or not with DOX and BNS-DOX for 72 h and the cell cycle was then assessed by flow cytometry. Graphs represented the % of the quantification of cell cycle phases from three independent experiments.

In order to assess whether the effects on the cell viability are related to inhibition of cell cycle progression, a cell cycle analysis was performed after 72 h of culture in the presence and absence of titrated amounts of DOX or BNS-DOX (Figure 3).

The different cell lines showed distinct patterns of cell cycle distribution and BNS-DOX or DOX treatments showed variable effects, but the most consistent one was an increase of subG1 cells, induced to a higher extent by BNS-DOX compared to DOX.

**Figure 4:**
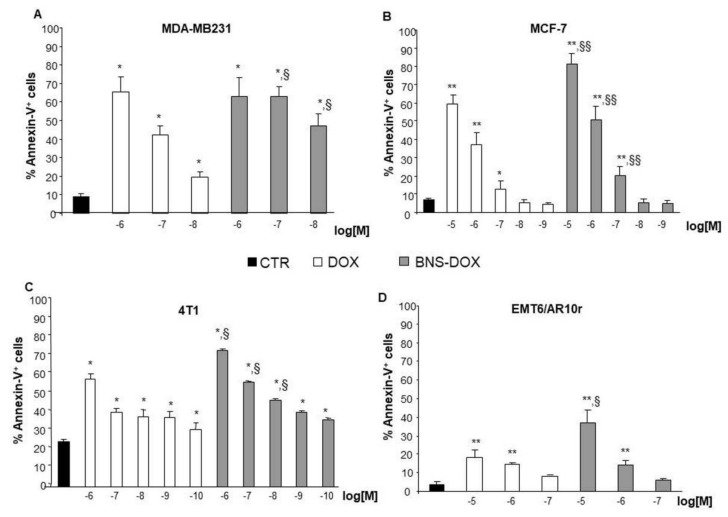
Effects of DOX and BNS-DOX treatment on cell death. Annexin-V positive cells were evaluated in (**A**,**B**) MDA-MB231 and MCF-7 human breast cancer cell lines and (**C**,**D**) 4T1 and EMR6/AR10r mouse breast cancer cell lines cultured for 72 h in the presence or absence of DOX or BNS-DOX. Results are expressed as % of positive cells from five independent experiments (* *p* < 0.05, ** *p* < 0.01, versus the control; ^§^ *p* < 0.05, ^§§^ *p* < 0.01, versus the same concentration).

These data suggest that the inhibition of cell growth induced by DOX or BNS-DOX can be due to induction of apoptosis. To verify this possibility, cells cultured for 72 h in the presence and absence of titrated amounts of DOX or BNS-DOX were stained with annexin-V and analyzed by flow cytometry to detect dying cells (Figure 4).

Results showed that treatment with DOX and BNS-DOX significantly increased the proportion of Annexin-V-positive cells, and BNS-DOX was significantly more effective than DOX in all cell lines, with patterns similar to those displayed by cell growth experiments.

**Figure 5:**
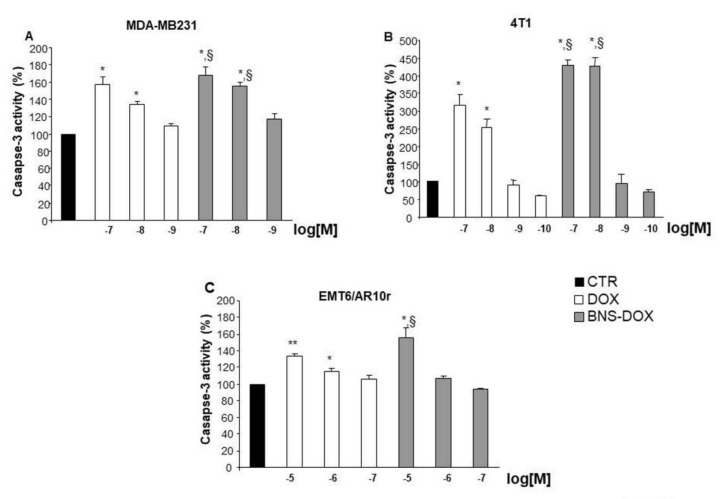
Levels of caspase3 activity after DOX and BNS-DOX treatment. Caspase-3 activity was evaluated in (**A**) MDA-MB231 human breast cancer cell lines and (**B**,**C**) 4T1 and EMR6/AR10r mouse breast cancer cell lines cultured for 72 h in the presence or absence of DOX or BNS-DOX. Results are expressed as % calculated as follows: (result displayed by each treatment/the results displayed by untreated cells) from five independent experiments (* *p* < 0.05, ** *p* < 0.01, versus the control; ^§^ *p* < 0.05 versus the same concentration).

To confirm the effect on cell apoptosis, we assessed caspase 3 activity on lysates of MDA-MB231, 4T1, and EMT6/AR10r cells cultured for 72 h in the presence and absence of titrated amounts of DOX or BNS-DOX. Results showed that both DOX and BNS-DOX activated caspase3 in all cell lines, and BNS-DOX was more effective than DOX (Figure 5).

**Figure 6:**
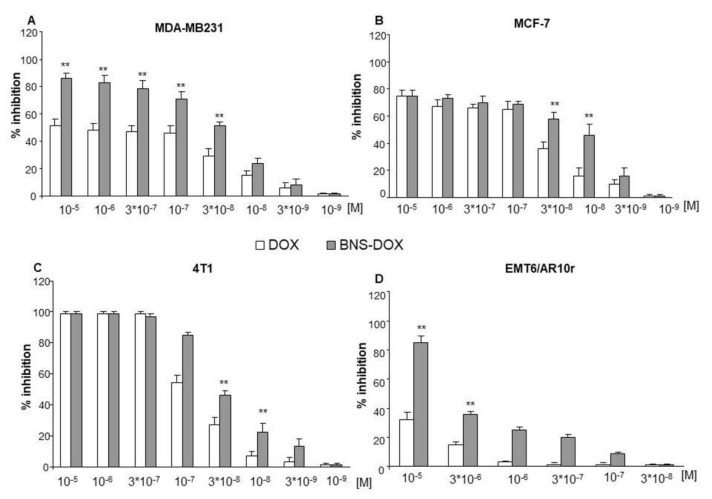
Effect of DOX and BNS-DOX on cell clonogenicity was tested by the colony forming assay. (**A**,**B**) MDA-MB231 and MCF-7 human breast cancer cell lines and (**C**,**D**) 4T1 and EMR6/AR10r mouse breast cancer cell lines were seeded in six-well plates and treated with each drug formulation at the indicated concentrations for 3 h. The medium was then changed and cells were cultured for an additional 10 days and subsequently fixed and stained with crystal violet. Graphs showed the % of clonogenicity inhibition (compared to controls) expressed as means ± SEM (*n* = 5). ** *p* < 0.01 significantly different from the same concentration of DOX.

Since MCF-7 cells do not express caspase3 [14,15] we did not evaluate its activity in this cell line. To further compare the effect of the two drug formulations on mammary cancer cell growth, we performed a clonogenic assay. Cells were treated for 3 h in the presence and absence of titrated amounts of BNS-DOX or DOX, then, the drug was removed and cells were cultured for 10 days. This approach was used in order to determine if the nanoparticles, once they penetrated inside the tumor cell, were able to function as a reservoir and release the drug during an extended period of time. Colony count at the end of the culture showed that, also in this case, BNS-DOX was more effective than free DOX in inhibiting cell growth in all cell lines (Figure 6).

**Figure 7:**
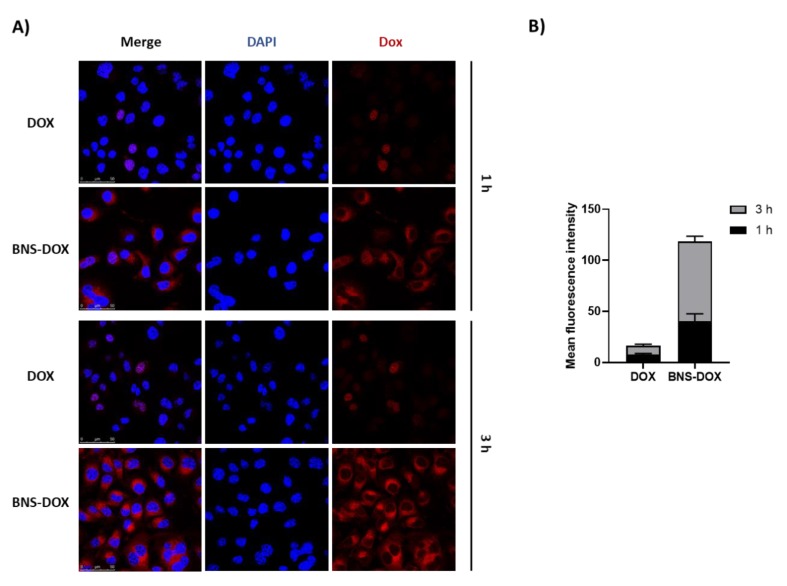
Cellular uptake studies. (**A**) BNS-DOX and DOX were incubated with EMT6 cells for 1 and 3 h. Exploiting doxorubicin (Dox) fluorescence, the cells were fixed and analyzed with Leica SPE confocal microscopy using a 63× oil lens. Images are representative of three field per condition (*n* = 3). (**B**) Quantification of Dox fluorescence inside the cells from images in panel A (*n*  =  3).

To evaluate the cellular uptake of BNS-DOX in EMT6 cells confocal microscopy studies were carried out, exploiting the intrinsic red fluorescence of doxorubicin. BNS-DOX were easily internalized in cells, in agreement with our previous studies. Figure 7A reports the confocal microscopy images after 1 h and 3 h of incubation, showing the localization of BNS-DOX in cell cytoplasm around the nucleus. In contrast, a lower mean fluorescence intensity was observed for the cells treated with free DOX (Figure 7B).

**Figure 8:**
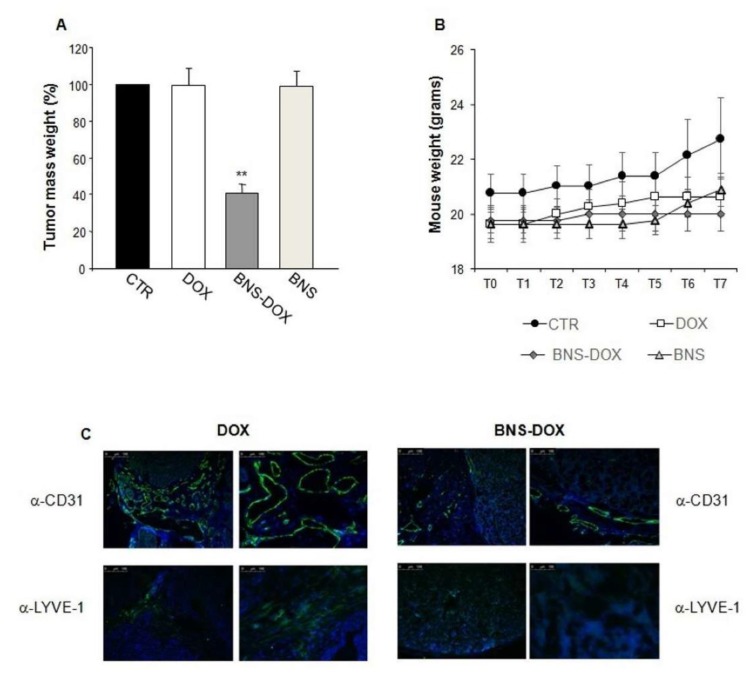
Effect of DOX and BNS-DOX on tumor growth in vivo. Thirteen-week-old female wt or BALB-neuT mice were treated with i.v. injection of 2 mg/kg DOX, or BNS-DOX, or BNS or the same volume of PBS as a control for six weeks (once every week). Mice were sacrificed at the seventh week after the first injection. (**A**) The graph shows an average of the tumor mass weight, expressed as % compared to the control group. (**B**) Weight of the animals was evaluated every week from the day of the first injection, performed at T0 (when the tumor was palpable); each treatment involved 8 mice. (**C**) Immunofluorescence staining of cluster of differentiation 31 (CD31) and lymphatic vessel endothelial hyaluronan receptor 1 (LYVE-1) of tumour tissue sections from of Neu-T mice treated with DOX or BNS-DOX. The slides were stained with either pAb rabbit α-mouse CD31 or pAb rabbit α-mouse LYVE-1 plus a secondary antibody α-rabbit conjugated with Alexa Fluor^®^ 488. Representative images of three independent experiments are shown (scale bar 100 µm). ** *p* < 0.01.

To compare the effect of DOX and BNS-DOX on breast cancer cell growth, we used the BALB-neuT mice, which has a strong similarity with the human HER2-positive tumors. BALB-neuT females develop spontaneous breast tumors with 100% penetrance and a known kinetic: at 60 days from birth (9 week) they develop in situ carcinoma, at 120–150 days invasive tumors, and at 230 days cancerous extensive tumor masses involving all mammary glands [13,16,17,18,19]. The treatment with DOX and BNS-DOX began at 16–18 weeks from birth and mice were treated weekly with intravenous injections of DOX, BNS-DOX, empty nanoparticles (BNS), or PBS. The animal weight was of about 20 grams at time 0 (Figure 8B). At sacrifice, after 7 weeks from the beginning of the treatment, the tumor weights of mice treated with BNS-DOX were about 60% lower than those of mice treated with either PBS, DOX, or empty BNS (Figure 8A).

To assess the effect of treatments on tumor neoangiogenesis and lymphangiogenesis, we analyzed the expression of CD31 marking blood vessels and LYVE-1 marking lymphoid vessels by immunofluorescence in cryostat sections cut in OCT, which allows optimal retention of the structural micro-architecture. Results showed that tumors from mice treated with BNS-DOX and DOX displayed substantially fewer areas of active angiogenesis and lymphatic vasculature than the tumors from mice treated with DOX (Figure 8C).

**Figure 9:**
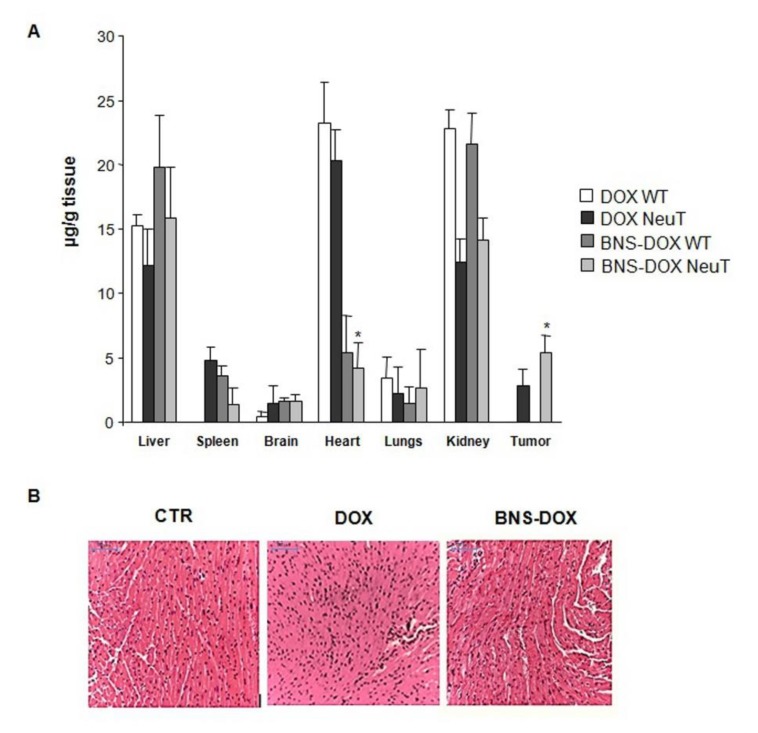
(**A**) Tissue biodistribution of DOX (expressed as µg/g tissue) after i.v. administration to BALB-neuT and BALB mice of 2 mg/kg of DOX or BNS-DOX. (**B**) Histopathologic analysis of the heart. Hematoxylin and eosin (H&E) stained were observed with 20× magnification (scale bar 100 µm). Representative image of heart tissue from Neu-T mice treated with phosphate buffered saline (PBS), DOX, or BNS-DOX. * *p* < 0.05.

Since DOX is highly lipophilic and systemically distributes among all tissues, we compared the tissue biodistribution of doxorubicin in the tumors and several organs of BALB-neuT mice treated with either BNS-DOX or DOX at sacrifice by HLPC analysis. Analysis of the tumors showed that those from mice treated with BNS-DOX contained significantly more doxorubicin than those tumors from mice treated with DOX. Analysis of the organs showed that mice treated with BNS-DOX and DOX did not display substantial differences in the drug content of lungs, liver, kidneys, spleen, and brain, whereas the drug content of the heart was significantly lower (5 times lower) in BNS-DOX mice than in DOX mice (Figure 9A).

Similar results were obtained by analyzing DOX distribution in control wild type BALB/c mice (not developing mammary tumors) receiving the same BNS-DOX and DOX treatment as BALB/neuT mice. In both BALB/c and BALB/neuT, treatment with BNS and DOX did not affect the weights of mice and organs compared with those of untreated mice.

Since these data suggest that BNS-DOX might be less cardiotoxic than DOX, we analyzed tissue sections stained with hematoxylin and eosin (H&E) staining obtained from the heart of mice treated with either BNS-DOX or DOX. Microscopic analysis showed that BNS-DOX did not affect the integrity and histology of the heart tissue, whereas DOX caused cardiotoxicity characterized by necrosis with red blood cells extravasation and moderate eosinophil infiltration (Figure 9B).

## 3. Discussion

DOX is widely used as a chemotherapy agent for the treatment of breast cancer [1]. Its cytotoxic and antiproliferative effects are exerted through several mechanisms, but the best known is in the poisoning of topoisomerases II cleavage complexes [20,21], causing G1 and G2 cell cycle arrest and induction of apoptosis. Other effects of DOX include its intercalation into DNA and the generation of free radicals causing DNA damage and lipid peroxidation [22].

Lack of specificity with inability to discriminate between malignant and noncancerous cells are the main causes of the side effects typical of standard DOX formulations, which limit doses and effectiveness of the drug. Moreover, DOX (or its derivatives) use is limited by cardiac and kidney toxicity and frequent onset of drug resistance in numerous tumor cell types [1]. In breast cancer cells, emergence of multidrug resistance (MDR) mainly involves upregulation of efflux transporters that are either located in the plasma or the nuclear membrane, which decrease the intracellular drug concentration [23].

Among them, the most extensively studied MDR transporters include P-glycoprotein (P-gp/ABCB1), multidrug resistance protein-1 (MRP1/ABCC1), and breast cancer resistance protein (BCRP, ABCG2) [24], all involved in drug inactivation and extrusion. Another possible feature of cancer cells is ability to escape apoptosis, caused by alterations in the apoptosis pathways [25,26].

To overcome these problems, several delivery systems have been proposed as carriers for DOX, but only liposomal DOX (Doxil^®^) achieved FDA approval in 1995 for treatment of Kaposi’s sarcoma [27]. Although Doxil^®^ is able to decrease cardiotoxicity in metastatic breast cancer patients [28], this formulation presents some limitations, such as fixed functionality, leakage of encapsulated agent, and high cost of manufacturing [29]. Moreover, although Doxil^®^ improves the pharmacokinetic properties of DOX, it does not increase the pharmacological efficacy of DOX because of the insufficient cell uptake of liposomes [30]. In this context, other types of DOX nanocarriers have been proposed [31], such as poly(lactic-co-glycolic acid) (PLGA) nanoparticles [32], solid lipid nanoparticles [33] or nanosponges with different structures [34,35,36,37].

Here, we investigated a new type of β-cyclodextrin nanosponges for the in vitro and in vivo delivery of doxorubicin. The doxorubicin BNS formulation prepared for this study is capable of incorporating a good amount of drug, forming a reservoir which allows the DOX release in a prolonged manner. Notably, DOX incorporation in the polymer nanoparticles can be attributed to hydrophilic and hydrophobic interactions. It is worth noting that nanosponges present multiple domains, i.e., the hydrophobic β-CD cavities and the hydrophilic nanochannels in the polymer network. Moreover, further electrostatic interactions between DOX amino groups and carboxylic groups of polymer matrix may occur. This nanostructure feature can increase the effectiveness of the drug loading. The loading capacity obtained in this study (16.4 w%) was in line with other nanosponge formulations previously designed by our group [36,37]. This result is particularly promising taking into account also the DOX incorporation in other kinds of nanocarriers reported in literature [33,38,39,40,41] Therefore, DOX-BNS was further investigated both in vitro and in vivo.

In vitro studies showed the physical stability of BNS-DOX aqueous nanosuspension in plasma without aggregation phenomena. The in vivo physical stability and safety of BNS-DOX after i.v. administration are two key parameters to be taken into account for a nanoformulation [42]. As far as concern the cytotoxicity, no toxic effects were observed with empty BNS.

Our in vitro experiments showed that BNS-DOX displays increased ability to decrease the growth of both the ER/PR+ and the TNBC cell lines, and even of the DOX-resistant EMT6/AR10r cells, compared to the free DOX. The similar efficacy between DOX and BNS-DOX, that was observed in 4T1 cells at higher drug concentrations, was probably linked to the relatively high sensitivity of this cell line to DOX treatment. This observation was in agreement with the higher number of SubG1 cells found in 4T1 DOX-treated cells. The proportion of subG1 cells after DOX treatment depended on the sensitivity of the different cell lines. Similar results were obtained in MCF-7 and MCF-7 DOX-resistant human breast cancer cells by De et al. 2014 [43]. Moreover, results obtained from the clonogenic assay demonstrated that a short term exposure of cells to BNS-DOX is sufficient to elicit subsequent inhibition of colony formation, possibly because of a rapid internalization of the drug into the cancer cells, followed by sustained release and decreased extrusion by MDR mechanisms, such as the energy-dependent Pg-p type efflux pumps which decrease the drug concentration in the cell and its effectiveness. Clonogenic experiments showed that DOX inhibited cell proliferation at higher doses, because it can enter inside the cells, thanks to its lipophilic characteristics, but it can easily extruded by the cells by MDR mechanism. Carcinoma of colon, kidney, pancreas, and liver express high P-gp levels, while lung, ovary, and breast carcinoma, melanoma, and lymphomas express low P-gp levels, which often increase after chemotherapy causing acquired drug resistance [44]. Probably, BNS-DOX are internalized through endocytosis [45] and DOX is then slowly released in the cytoplasm far from these efflux pumps, reducing drug elimination. Indeed, cell uptake studies by confocal microscopy showed the internalization of BNS-DOX into the cells and their localization in the cytoplasm around the cell nucleus. Therefore, BNS-DOX could act as a drug reservoir inside the cells, that slowly release the drug.

The inhibitory effect on cell growth are mainly ascribable to induction of cell apoptosis, as indicated by the increase of the sub-G1 cell population detected by cell cycle analysis, of the proportion of dying cells stained by AnnexinV and of capsase3 activation.

The in vivo studies confirm the higher anticancer effect of BNS-DOX compared to DOX. Particularly relevant was the choice of the murine BALB-neuT model, which recapitulates several features of human tumors displaying mutations of the *ERBB2* proto-oncogene coding for receptor tyrosine-protein kinase erbB-2, also known as *HER2* (from human epidermal growth factor receptor 2) or *HER2/neu* [46]. HER2 is a member of the epidermal growth factor receptors (EGFR) family that has a crucial role in cell proliferation, apoptosis, adhesion, and cellular death; a mutation or an over expression of this gene can lead to carcinogenesis [47]. Results show that BNS-DOX reduces by 60% the tumor growth compared to DOX, at a 2 mg/kg dose that is 5-fold lower than the DOX therapeutic one (10 mg/kg). Indeed, at this dose, the free DOX is almost ineffective. This outcome might be related to the enhanced permeability and retention (EPR) effect. The irregular formation of blood and lymphatic vessels in the tumor, favored the extravasation and entrapment of the nanoparticles in the target site. In line with this possibility, our analysis of drug biodistribution detected higher levels of doxorubicin in the tumors of the mice treated with BNS-DOX than in those treated with DOX.

Besides these effects, the increased therapeutic effect of BNS-DOX may be partly ascribed to inhibition of tumor neoangiogenesis and lymphangiogenesis, detected by the immunohistological analysis of cancer tissue sections, which would limit nutrients and oxygen available to the tumor, and also tumor metastatic spread. Moreover, decreased lymphangiogenesis would favor entrapment of BNS-DOX in the tumor. This behavior was already observed with paclitaxel loaded in BNS in melanoma cell models [48].

Furthermore, the in vivo experiments did not detect severe toxic side effects of BNS-DOX, as a matter of fact it did not affect the aspect of the fur, weight, consumption of food and water, and viability of the animals. Interestingly, the drug biodistribution analysis showed drug levels in heart tissue of mice treated with BNS-DOX are 5-fold lower than those detected in mice treated with DOX, which may be relevant in decreasing the cardiotoxic side effects of DOX, being this effect the main limitation of the drug. In line with this observation, histological analysis of heart tissue sections detected signs of cardiac damage in the DOX-treated mice, but not in those treated with BNS-DOX. A further point is that, in vitro too, BNS-DOX display less toxicity than DOX on cultures of rat cardiomyocytes. The low level of heart accumulation might depend on the size (about 300 nm of diameter) and chemical structure of our BNS, capillary filtration and modality of internalization into cells [49]. This capability of doxorubicin loaded in nanosponges to reduce the cardiotoxicity was already observed with other nanosponge formulations [37].

Taken together, all these results underline the promising features of BNS-DOX to improve the therapeutic outcome of the drug.

## 4. Materials and Methods

### 4.1. Materials

The β-CDs were a kind gift from Roquette Italia (Cassano Spinola, Italy). All reagents were of analytical grade. The laboratory reagents were from Sigma-Aldrich, unless otherwise specified. The cell culture reagents were purchased from Gibco/Invitrogen (Life Technologies, Paisley, UK), unless otherwise indicated.

### 4.2. Preparation of Doxorubicin-Loaded Nanosponges

BNS were synthetized using β-cyclodextrins as building block and pyromellitic anhydride as crosslinking agent in 1:4 molar ratio (CD/cross-linker). The synthesis was carried out according our previous works [48,50].

A blank BNS aqueous nanosuspension (10 mg/mL) was prepared by homogenizing the BNS coarse powder suspended in saline solution (NaCl 0.9% *w*/*v*) firstly with a high-shear homogenizer (Ultra-Turrax, IKA, Konigswinter, Germany, 10 min, 24,000 rpm) and then with a high-pressure homogenizer (EmulsiFlex C5, Avastin, Mannheim, Germany, 90 min, 500 bar) to further reduce the BNS size.

Then, doxorubicin was added to the blank BNS nanosuspension to obtain doxorubicin loaded BNS (BNS-DOX) at a concentration of 2 mg/mL. The sample was incubated under stirring for 24 h at room temperature in the dark, to promote the loading. A dialysis step was performed to eliminate the unloaded doxorubicin.

### 4.3. Characterization of Doxorubicin-Loaded Nanosponges

BNS and BNS-DOX samples were in vitro characterized by determining their average diameter, polidispersity index, and zeta potential with dynamic light scattering (90 Plus particle sizer, Brookhaven Instruments Corporation, Holtsville, NY, USA). The samples were diluted with water (1:30 *v*/*v*) immediately prior the measurements, carried out at a fixed angle of 90° and at a temperature of 25 °C. Transmission electron microscopy (TEM) analysis was performed using a Philips CM 10 transmission electron microscope to observe the morphology of BNS-DOX. The BNS nanosuspensions were sprayed on Formvar coated copper grid and air-dried before observation.

A weighted amount of freeze-dried BNS-DOX was dispersed in 5 mL of water and sonicated for 15 min. The sample was then centrifuged (10 min, 15,000 rpm) and after the supernatant was analyzed by High Performance Liquid Cromatography (HPLC), to determine the DOX concentration in the BNS.

The loading capacity of BNS-DOX was calculated according to: Loading capacity (%) = [amount of DOX/weight of BNS-DOX] × 100. The encapsulation efficiency of BNS-DOX was determined using the formula: Encapsulation efficiency (%) = [amount of DOX loaded/total amount of DOX] × 100.

The physical stability of BNS-DOX stored at 4 °C was assessed up to 1 month, by checking the physicochemical parameters over time.

### 4.4. In Vitro Release Studies

The in vitro release kinetics of DOX from BNS-DOX was evaluated with a multi-compartment rotating cell, using a cellulose membrane (Spectrapore, cut-off = 12,000 Da) to separate the donor from the receiving compartment. The in vitro drug release studies were performed at two different pH values (5.5 and 7.4). Samples of receiving phase (phosphate buffered saline, PBS at pH 5.5 or 7.4) were withdrawn at fixed times and analyzed by HPLC to quantify the amount of drug released from BNS-DOX over time. DOX was detected using a fluorescence detector at excitation and emission wavelengths of 480 and 560 nm, respectively.

### 4.5. Stability and In Vitro Release Studies in Plasma

The stability of BNS-DOX was investigated in plasma, determining their size and DOX concentration over time after BNS-DOX incubation in plasma at 37 °C for 24 h. The in vitro release kinetics of DOX from BNS-DOX in plasma was also studied. For this purpose 50 microliters of BNS-DOX were incubated at 37 °C with rat plasma diluted with PBS at pH = 7.4 (1:3 *v*/*v*). At fixed time 200 microliters of the sample were withdrawn, centrifuged using a centrifugal filter device (Amicon^®^ Ultra, 20,000 rpm, 10 min) and then the filtrate was analyzed by HPLC to quantify the amount of DOX released from BNS-DOX over time.

### 4.6. Cell Culture Conditions

Cancer cell lines were obtained from the American Type Culture Collection (ATCC; Manassas, VA, USA). MDA-MB231, MCF-7, H9c2 were grown in culture dishes as a monolayer in Dulbecco’s Modified Eagle (DMEM) medium (Invitrogen, Burlington, ON, Canada), 4T1 in RPMI 1640, and EMT6/AR10r DOX resistant in Minimum Essential Medium (MEM), all supplemented with 10% penicillin-streptomycin (Invitrogen) and 10% fetal calf serum (Invitrogen), in a humidified atmosphere with 5% CO_2_. In the case of EMT6/AR10r DOX resistant cell lines, 1 μg/mL of DOX was added in the culture medium once a week.

### 4.7. Cell Proliferation

MTT (3-(4,5-Dimethylthiazol-2-yl)-2,5diphenyltetrazolium bromide; Sigma-Aldrich, St. Louis, MA, USA) analysis was performed in 96-well plates. Briefly, 1000–2000 cells/well were seeded in 100 µL of complete medium and the day after they were treated with DOX or BNS-DOX (10^−5^–10^−9^ M) for 72 h. Subsequently, cells were supplemented with 5 mg/mL thiazolyl blue tetrazolium bromide (M2128; Sigma-Aldrich) for 2 h. Thereafter, the medium was removed and cells were lysed with 100 µL of DMSO. Absorbance was recorded at 570 nm by a 96-well-plate reader (PerkinElmer, Waltham, MA, USA). Controls were normalized to 100%, and the readings from treated cells were expressed as % of viability inhibition. Eight replicates were used to determine each data point, and five different experiments were performed.

### 4.8. Colony-Forming Assay

Cells (800/well) were seeded into six well plates and treated with the compounds. The medium was changed after 3 h and cells were cultured for additional 10 days. Subsequently, cells were fixed and stained with a solution of 80% crystal violet and 20% methanol. Colonies were then photographed and counted with a Gel Doc equipment (Bio-Rad Laboratories, Hercules, CA, USA). Then, cells were washed and 30% acetic acid were added to induce a complete dissolution of the crystal violet. Absorbance was recorded at 595 nm by a 96-well-plate ELISA reader. Controls were normalized to 100%, and the readings from treated cells were expressed as % of viability inhibition. Five different experiments were performed.

### 4.9. Cell Cycle Analysis

Treated and untreated cells were harvested 72 h after the treatment with DOX or BNS-DOX at different concentrations. Cells were washed with 1× PBS, fixed in 70% cold ethanol, resuspended in a buffer containing 0.02 mg/mL RNase A (Sigma-Aldrich), 0.05 mg/mL propidium iodide (Sigma-Aldrich), 0.2% *v*/*v* Nonidet P-40 (Sigma-Aldrich), 0.1% *w/v* sodium citrate (Sigma-Aldrich), and analyzed by a FACS Calibur cytometer (Becton Dickinson, Franklin Lakes, NJ, USA).

### 4.10. Cell Death

Treated and untreated cells were harvested 72 h after the treatment with different concentration of DOX or BNS-DOX, washed with 1× PBS, and subsequently resuspended in Annexin-V binding buffer (Life Technologies, Carlsbad, CA, USA) supplemented with 1:100 APC-conjugated Annexin-V (Immunotools, Friesoythe, Germany). Cells were analyzed by a FACS Calibur cytometer (Becton Dickinson, Franklin Lakes, NJ, USA). The analysis of apoptosis was performed by measuring the caspase 3 activity in cell lysates using a fluorimetric assay (BioVision, Milpitas, CA, USA) following the manufacture’ instructions.

### 4.11. Cell Uptake Studies

EMT6 cells (4 × 10^4^ cells per well) were seeded on Corning^®^ cover glasses (Sigma-Aldrich) in a 24-well plate. The day after, the cells were incubated with BNS-DOX and DOX (30 µM) for 1 and 3 hours. Then, the cells were washed with PBS, fixed with 4% paraformaldehyde at room temperature for 15 min, and stained with 4′,6-diamidine-2-phenylindole (DAPI). Finally, coverslips were mounted and the image acquisition was performed with a Leica SPE Confocal microscope (Leica, Wetzlar, Germany), with 63X oil-immersion objective. The mean fluorescence intensity of Dox was quantified by ImageJ software (ImageJ, U.S. National Institutes of Health, Bethesda, MD, USA).

### 4.12. In Vivo Experiments

Transgenic (BALB-neuT) female mice, which over-express the rat HER2 (neu) oncogene under the mouse mammary tumor virus (MMTV) promoter [51] and wild type mice (BALB), 16–18 weeks old, were used. BALB-neuT and BALB mice were treated every week with intravenous injections (i.v.) of 2 mg/kg of DOX, BNS-DOX, empty nanoparticles (BNS), or PBS as control. Mice were bred under pathogen-free conditions in the animal facility of the Università del Piemonte Orientale, and treated in accordance with the Bioethics Committee of University of Turin, protocol NO. 479 of 1 November 2016. Body weight of the animals was monitored over time. Mice were sacrificed 7 weeks after the beginning of treatment and tumors were harvested and weighed. Immediately after dissection, tumor samples were embedded in OCT compound, snap-frozen and stored at −80 °C until use.

The biodistribution of DOX and BNS-DOXwas evaluated after the mice sacrifice. The different tissues (liver, spleen, brain, heart, lungs, and kidneys) were collected, washed with saline solution, weighed, and frozen at −80 °C until analysis. Tissue extracts were prepared by adding 200 µL of methanol and 400 µL of TRIS buffer (1 M, pH 8.5) to the tissue and then homogenizing the mixtures.

The tissue homogenates were kept in ice for 15 min before adding 1.4 mL of acetonitrile. Then, the samples were vortex and centrifuged for 5 min at 15,000 rpm to separate the precipitated proteins.

After centrifugation clear supernatants (100 µL) were analyzed by HPLC analysis, to quantify the amount of DOX in the tissue extracts.

### 4.13. Immunofluorescence

Tumor tissues were cut with a cryostat (thickness 5–6 µm) and treated with 4% paraformaldehyde (Sigma-Aldrich) diluted in PBS for 5 min at room temperature to fix the sample on the glass slides. The samples were then blocked with 5% normal goat serum (NGS) in PBS for 1 h, in order to block aspecific sites to which the primary antibody could bind. To detect CD31 and LYVE-1 expression, slides were incubated with the primary rabbit antibody anti-CD31 or anti-LYVE-1 (dilution 1:100 and 1:200, respectively; Abcam, Cambridge, UK) overnight at 4 °C. The secondary antibody used was an anti-rabbit Ig Alexa fluor 488-conjugated (Invitrogen), diluted 1: 400. Then the sections were stained with 0.5 mg/mL of the fluorescent dye 4,6-diamidino-2-phenylindole-dihydrochloride (DAPI, Sigma-Aldrich) for 5 min, to stain the cell nuclei, and then mounted using Prolong anti-fade mounting medium (Light AntiFADE Kit, Molecular Probes Invitrogen). The sections were then observed by a fluorescence inverted Axiovert 40 CFL microscope (Carl Zeiss, Oberkochen, Germany), photographed with a Retiga 200R digital camera (QImaging, Surrey, BC, Canada), and analyzed with the Image Pro Plus Software for micro-imaging 5.0 (Media Cybernetics, version 5.0, Bethesda, MD, USA).

### 4.14. Histopathological Examination

Transverse midsections of hearts were fixed in 10% buffered formalin, embedded in paraffin, cut into 5 μm sections, and processed for hematoxylin and eosin (H&E) staining to determine the level of inflammation. Stained sections were analyzed by light microscope (DM 2500 Leica) connected to a digital camera (DFC 320, Leica, Milan, Italy). The sections were morphologically examined to evaluate the inflammatory infiltration levels.

### 4.15. Data Analysis

Data are shown as mean ± SEM. The statistical analyses were performed with GraphPad Prism 3.0 software (San Diego, CA, USA) using the one-way ANOVA and Dunnett test. IC_50_ was investigated using the two tailed unpaired *t*-test with Welch correction. The significance cut-off was *p* below 0.05.

## 5. Conclusions

BNS-DOX proved to be a promising effective nanoformulation in the treatment of breast cancer. The increased effectiveness, compared to DOX, may be due to a higher accumulation into the tumor mass and the neoplastic cells. Moreover, the reduced biodistribution in the heart tissue suggests a reduced cardiotoxicity of BNS-DOX. Therefore, this nanoformulation, which increases DOX efficacy and decreases its severe side effects, might be an attractive tool for improving the clinical management of breast cancer.

## Figures and Tables

**Table 1:** Physico-chemical characteristics of blank and doxorubicin (DOX)-loaded β-cyclodextrin nanosponges (BNS).

Formulation	Average Diameter ± SD (nm)	Polydispersity Index	Zeta Potential ± SD (mV)
Blank BNS	302.5 ± 4.6	0.20 ± 0.01	−35.2 ± 2.7
BNS-DOX	310.4 ± 5.7	0.21 ± 0.01	−29.8 ± 1.3

**Table 2:** Half maximal inhibitory concentration (IC_50_) of BNS-DOX and DOX.

Cell Line	IC_50_ BNS-DOX	IC_50_ DOX	*p*-Value
MDA-MB231	1.6 ± 0.2 × 10^−7^	5 ± 0.2 × 10^−7^	0.001
4T1	2.8 ± 0.2 × 10^−8^	8.64 ± 0.8 × 10^−8^	0.0021
EMT6/AR10r	5.3 ± 0.67 × 10^−6^	29 ± 0.9 × 10^−5^	0.0045
MCF-7	1.5 ± 0.5 × 10^−8^	15 ± 0.9 × 10^−7^	0.001

Two-tailed *p*-value has been determined with unpaired *t*-test with Welch correction.

## Abbreviations

DOX	doxorubicin
BNS-DOX	*β*-Cyclodextrins Nanosponges containing doxorubicin
EPR	enhanced permeability and retention effect
CD	cyclodextrin
BNS	β-cyclodextrin nanosponges
TBNC	Triple negative cell-lines
P-gp	P-glycoprotein
PLGA	poly (lactic-co-glycolic acid)
EGFR	epidermal growth factor receptors
MTT	(3-(4,5-Dimethylthiazol-2-yl)-2,5diphenyltetrazolium bromide
MMTV	mouse mammary tumor virus
NGS	normal goat serum
H&E	hematoxylin and eosin

## References

[1] Humber C.E., Tierney J.F., Symonds R.P., Collingwood M., Kirwan J., Williams C., Green J.A. (2007). Chemotherapy for advanced, recurrent or metastatic endometrial cancer: A systematic review of Cochrane collaboration. Ann. Oncol..

[2] Prasad P., Shuhendler A., Cai P., Rauth A.M., Wu X.Y. (2013). Doxorubicin and mitomycin C-loaded polymer-lipid hybrid nanoparticles inhibit growth of sensitive and multidrug resistant human mammary tumor xenografts. Cancer Lett..

[3] Jeong Y.I., Chung K.D., Choi K.C. (2011). Doxorubicin release from self-assembled nanoparticles of deoxycholic acid-conjugated dextran. Arch. Pharm. Res..

[4] Hobbs S.K., Monsky W.L., Yuan F., Roberts W.G., Griffith L., Torchilin V.P., Jain R.K. (1998). Regulation of transport pathways in tumor vessels: Role of tumor type and microenvironment. Proc. Natl. Acad. Sci. USA.

[5] Laza-Knoerr A.L., Gref R., Couvreur P. (2010). Cyclodextrins for drug delivery. J. Drug Targets.

[6] Gigliotti C.L., Minelli R., Cavalli R., Occhipinti S., Barrera G., Pizzimenti S., Cappellano G., Boggio E., Conti L., Fantozzi R. (2016). In Vitro and In Vivo therapeutic evaluation of camptothecin-encapsulated β-cyclodextrin nanosponges in prostate cancer. J. Biomed. Nanotechnol..

[7] Argenziano M., Lombardi C., Ferrara B., Trotta F., Caldera F., Blangetti M., Koltai H., Kapulnik Y., Yarden R., Gigliotti L. (2018). Glutathione/pH-responsive nanosponges enhance strigolactone delivery to prostate cancer cells. Oncotarget.

[8] Duchene D., Cavalli R., Gref R. (2016). Cyclodextrin-based polymeric nanoparticles as efficient carriers for anticancer drugs. Curr. Pharm. Biotechnol..

[9] Swaminathan S., Pastero L., Serpe L., Trotta F., Vavia P., Aquilano D., Trotta M., Zara G., Cavalli R. (2010). Cyclodextrin-based nanosponges encapsulating camptothecin: Physicochemical characterization, stability and cytotoxicity. Eur. J. Pharm. Biopharm..

[10] Swaminathan S., Vavia P.R., Trotta F., Cavalli R., Tumbiolo S., Bertinetti L., Coluccia S. (2013). Structural evidence of differential forms of nanosponges of betacyclodextrin and its effect on solubilization of a model drug. J. Incl. Phenom. Macrocycl. Chem..

[11] Trotta F., Zanetti M., Cavalli R. (2012). Cyclodextrin-based nanosponges as drug carriers. Beilstein J. Org. Chem..

[12] Swaminathan S., Cavalli R., Trotta F. (2016). Cyclodextrin-based nanosponges: A versatile platform for cancer nanotherapeutics development. Wiley Interdiscip. Rev. Nanomed. Nanobiotechnol..

[13] Conti L., Ruiu R., Barutello G., Macagno M., Bandini S., Cavallo F., Lanzardo S. (2014). Microenvironment, oncoantigens, and antitumor vaccination: Lessons learned from BALB-neuT mice. Biomed. Res. Int..

[14] Jänicke R.U. (2009). MCF-7 breast carcinoma cells do not express caspase-3. Breast Cancer Res. Treat..

[15] Kagawa S., Gu J., Honda T., McDonnell T.J., Swisher S.G., Roth J.A., Fang B. (2001). Deficiency of caspase-3 in MCF7 cells blocks Bax-mediated nuclear fragmentation but not cell death. Clin. Cancer Res..

[16] Calogero R.A., Cordero F., Forni G., Cavallo F. (2007). Inflammation and breast cancer. Inflammatory component of mammary carcinogenesis in ErbB2 transgenic mice. Breast Cancer Res..

[17] Di Carlo E., Diodoro M.G., Boggio K., Modesti A., Modesti M., Nanni P., Forni G., Musiani P. (1999). Analysis of mammary carcinoma onset and progression in HER-2/neu oncogene transgenic mice reveals a lobular origin. Lab. Investig..

[18] Iezzi M., Calogero R.A., Spadaro M., Musiani P., Forni G., Cavallo F. (2012). BALB-neuT female mice as a dynamic model of mammary cancer. Bentham Sci. Publ. Transl. Anim. Models Drug Discov. Dev..

[19] Rovero S., Amici A., Di Carlo E., Bei R., Nanni P., Quaglino E., Porcedda P., Boggio K., Smorlesi A., Lollini P.L. (2000). DNA vaccination against rat her-2/Neu p185 more effectively inhibits carcinogenesis than transplantable carcinomas in transgenic BALB/c mice. J. Immunol..

[20] McClendon A.K., Osheroff N. (2007). DNA topoisomerase II, genotoxicity, and cancer. Mutat. Res..

[21] Tewey K.M., Rowe T.C., Yang L., Halligan B.D., Liu L.F. (1984). Adriamycin-induced DNA damage mediated by mammalian DNA topoisomerase II. Science.

[22] Minotti G., Menna P., Salvatorelli E., Cairo G., Gianni L. (2004). Anthracyclines: Molecular advances and pharmacologic developments in antitumor activity and cardiotoxicity. Pharmacol. Rev..

[23] Chang G. (2003). Multidrug resistance ABC transporters. FEBS Lett..

[24] Shafei A., El-Bakly W., Sobhy A., Wagdy O., Reda A., Aboelenin O., Marzouk A., El Habak K., Mostafa R., Ali M.A. (2017). A review on the efficacy and toxicity of different doxorubicin nanoparticles for targeted therapy in metastatic breast cancer. Biomed. Pharmacother..

[25] Kim T.H., Shin Y.J., Won A.J., Lee B.M., Choi W.S., Jung J.H., Chung H.Y., Kim H.S. (2014). Resveratrol enhances chemosensitivity of doxorubicin in multidrug-resistant human breast cancer cells via increased cellular influx of doxorubicin. Biochim. Biophys. Acta.

[26] Ruiz de Almodóvar C., Ruiz-Ruiz C., Muñoz-Pinedo C., Robledo G., López-Rivas A. (2001). The differential sensitivity of Bc1-2-overexpressing human breast tumor cells to TRAIL or doxorubicin-induced apoptosis is dependent on Bc1-2 protein levels. Oncogene.

[27] Barenholz Y. (2012). Doxil^®^—The first FDA-approved nano-drug: Lessons learned. J. Control. Release.

[28] O’Brien M.E., Wigler N., Inbar M., Rosso R., Grischke E., Santoro A., Catane R., Kieback D.G., Tomczak P., Ackland S.P. (2004). Reduced cardiotoxicity and comparable efficacy in a phase III trial of pegylated liposomal doxorubicin HCl (CAELYX/Doxil) versus conventional doxorubicin for first-line treatment of metastatic breast cancer. Ann. Oncol..

[29] Adair J.H., Parette M.P., Altinoğlu E.I., Kester M. (2010). Nanoparticulate alternatives for drug delivery. ACS Nano.

[30] Moosavian S.A., Abnous K., Badiee A., Jaafari M.R. (2016). Improvement in the drug delivery and anti-tumor efficacy of PEGylated liposomal doxorubicin by targeting RNA aptamers in mice bearing breast tumor model. Colloids Surf. B Biointerfaces.

[31] Gonçalves M., Mignani S., Rodrigues J., Tomás H. (2020). A glance over doxorubicin based-nanotherapeutics: From proof-of-concept studies to solutions in the market. J. Control. Release.

[32] Acharya S., Sahoo S.K. (2011). PLGA nanoparticles containing various anticancer agents and tumour delivery by EPR effect. Adv. Drug Deliv. Rev..

[33] Fundarò A., Cavalli R., Bargoni A., Vighetto D., Zara G.P., Gasco M.R. (2000). Non-stealth and stealth solid lipid nanoparticles (SLN) carrying doxorubicin: Pharmacokinetics and tissue distribution after iv administration to rats. Pharmacol. Res..

[34] Trotta F., Dianzani C., Caldera F., Mognetti B., Cavalli R. (2014). The application of nanosponges to cancer drug delivery. Expert Opin. Drug Deliv..

[35] Cavalli R., Trotta F., Tumiatti W. (2006). Cyclodextrin-based nanosponges for drug delivery. J. Incl. Phenom. Macrocycl. Chem..

[36] Caldera F., Argenziano M., Trotta F., Dianzani C., Gigliotti L., Tannous M., Pastero L., Aquilano D., Nishimoto T., Higashivama T. (2018). Cyclic nigerosyl-1, 6-nigerose-based nanosponges: An innovative pH and time-controlled nanocarrier for improving cancer treatment. Carbohydr. Polym..

[37] Daga M., Ullio C., Argenziano M., Dianzani C., Cavalli R., Trotta F., Ferretti C., Zara G.P., Gigliotti C.L., Ciamporcero E.S. (2016). GSH-targeted nanosponges increase doxorubicin-induced toxicity “in vitro” and “in vivo” in cancer cells with high antioxidant defenses. Free Radic. Biol. Med..

[38] Scomparin A., Salmaso S., Eldar-Boock A., Ben-Shushan D., Ferber S., Tiram G., Shmeeda H., Landa-Rouben N., Leor J., Caliceti P. (2015). A comparative study of folate receptor-targeted doxorubicin delivery systems: Dosing regimens and therapeutic index. J. Control. Release.

[39] Sun H., Guo X., Zeng S., Wang Y., Hou J., Yang D., Zhou S. (2019). A multifunctional liposomal nanoplatform co-delivering hydrophobic and hydrophilic doxorubicin for complete eradication of xenografted tumors. Nanoscale.

[40] Wang H., Zheng M., Gao J., Wang J., Zhang Q., Fawcett J.P., He Y., Gu J. (2020). Uptake and release profiles of PEGylated liposomal doxorubicin nanoparticles: A comprehensive picture based on separate determination of encapsulated and total drug concentrations in tissues of tumor-bearing mice. Talanta.

[41] Marano F., Argenziano M., Frairia R., Adamini A., Bosco O., Rinella L., Fortunati N., Cavalli R., Catalano M.G. (2016). Doxorubicin-loaded nanobubbles combined with extracorporeal shock waves: Basis for a new drug delivery tool in anaplastic thyroid cancer. Thyroid.

[42] Yang Y., Yang Y., Xie X., Cai X., Zhang H., Gong W., Wang Z., Mei X. (2014). PEGylated liposomes with NGR ligand and heat-activable cell-penetrating peptide-doxorubicin conjugate for tumor-specific therapy. Biomaterials.

[43] De U., Chun P., Choi W.S., Lee B.M., Kim N.D., Moon H.R., Jung J.H., Kim H.S. (2014). A novel anthracene derivative, MHY412, induces apoptosis in doxorubicin-resistant MCF-7/Adr human breast cancer cells through cell cycle arrest and downregulation of P-glycoprotein expression. Int. J. Oncol..

[44] Kapse-Mistry S., Govender T., Srivastava R., Yergeri M. (2014). Nanodrug delivery in reversing multidrug resistance in cancer cells. Front. Pharmacol..

[45] Rosenbaum A.I., Zhang G., Warren J.D., Maxfield F.R. (2010). Endocytosis of beta-cyclodextrins is responsible for cholesterol reduction in Niemann-Pick type C mutant cells. Proc. Natl. Acad. Sci. USA.

[46] Guy C.T., Webster M.A., Schaller M., Parsons T.J., Cardiff R.D., Muller W.J. (1992). Expression of the neu protooncogene in the mammary epithelium of transgenic mice induces metastatic disease. Proc. Natl. Acad. Sci. USA.

[47] Mitri Z., Constantine T., O’Regan R. (2012). The HER2 receptor in breast cancer: Pathophysiology, clinical use, and new advances in therapy. Chemother. Res. Pract..

[48] Clemente N., Argenziano M., Gigliotti C.L., Ferrara B., Boggio E., Chiocchetti A., Caldera F., Trotta F., Benetti E., Annaratone L. (2019). Paclitaxel-loaded nanosponges inhibit growth and angiogenesis in melanoma cell models. Front. Pharmacol..

[49] DeJong W.H., Hagens W.I., Krystek P., Burger M.C., Sips A.J., Geertsma R.E. (2008). Particle size-dependent organ distribution of gold nanoparticles after intravenous administration. Biomaterials.

[50] Clemente N., Boggio E., Gigliotti C.L., Raineri D., Ferrara B., Miglio G., Argenziano M., Chiocchetti A., Cappellano G., Trotta F. (2019). Immunotherapy of experimental melanoma with ICOS-Fc loaded in biocompatible and biodegradable nanoparticles. J. Control. Release.

[51] Quaglino E., Mastini C., Forni G., Cavallo F. (2008). ErbB2 transgenic mice: A tool for investigation of the immune prevention and treatment of mammary carcinomas. Curr. Protoc. Immunol..

